# Ambient Intelligence Application Based on Environmental Measurements Performed with an Assistant Mobile Robot

**DOI:** 10.3390/s140406045

**Published:** 2014-03-27

**Authors:** Dani Martinez, Mercè Teixidó, Davinia Font, Javier Moreno, Marcel Tresanchez, Santiago Marco, Jordi Palacín

**Affiliations:** 1 Department of Computer Science and Industrial Engineering, University of Lleida, Jaume II, 69, 25001 Lleida, Spain; E-Mails: dmartinez@diei.udl.cat (D.M.); mteixido@diei.udl.cat (M.T.); dfont@diei.udl.cat (D.F.); jmoreno@diei.udl.cat (J.M.); mtresanchez@diei.udl.cat (M.T.); 2 Department of Electronics, University of Barcelona, Martí Franquès, 1, 08028 Barcelona, Spain; 3 Signal and Information Processing for Sensing Systems, Institute for BioEngineering of Catalonia, Baldiri Reixac, 10-12, 08028 Barcelona, Spain; E-Mail: smarco@ibecbarcelona.eu

**Keywords:** ambient intelligence, human thermal comfort, robotic exploration

## Abstract

This paper proposes the use of an autonomous assistant mobile robot in order to monitor the environmental conditions of a large indoor area and develop an ambient intelligence application. The mobile robot uses single high performance embedded sensors in order to collect and geo-reference environmental information such as ambient temperature, air velocity and orientation and gas concentration. The data collected with the assistant mobile robot is analyzed in order to detect unusual measurements or discrepancies and develop focused corrective ambient actions. This paper shows an example of the measurements performed in a research facility which have enabled the detection and location of an uncomfortable temperature profile inside an office of the research facility. The ambient intelligent application has been developed by performing some localized ambient measurements that have been analyzed in order to propose some ambient actuations to correct the uncomfortable temperature profile.

## 1. Introduction

An ambient intelligence application [1,2] requires the combined use of sensors and actuators in order to respond to the actions and needs of the persons. For example, human health and productivity in an office is strongly correlated with the environmental working conditions and all efforts focused on improving the occupancy comfort will have a direct positive effect on the overall occupancy productivity [3], as it has been proved that when the temperature is above 25 °C the productivity decreases in 2% per degree [3]. In this particular application, the human thermal comfort can be evaluated with different indexes such as the predictive mean vote (PMV) index [4], that evaluates six key factors: air temperature, radiant temperature, humidity, air velocity, clothing level and activity of which the first four can be obtained by performing objective measurements with single and fixed individual sensors, fixed networks of sensors or with single sensors embedded in a mobile robot.

Another ambient intelligence application is the proposal of [5] were a wireless sensor network was used to detect arrhythmia to support medical decisions. In [6], a ZigBee sensor network was used to monitor physiological signals and send alarms to the family in case of any emergency. In [7], different sensing technologies were combined in order to develop a small kitchen. In [8], the Kinect^®^ depth sensor was used for automatic fall detection. In [8] the physical environment component of a home care monitoring system includes a sensor network and a telepresence mobile robot.

Alternatively, in [9] several mobile robots were used for mapping and fire detection. In [10], a survey of different algorithms based on simultaneous location and mapping for the development of 3D maps for urban search and rescue were presented. In [11], the passive RFID technology was used for mapping in order to develop a mobile security agent based on a multi-sensor robotic platform. In [12], a mobile robot was used to measure and map the local magnetic field in a corridor in order to detect anomalies and in [13] for odor source detection based on gas distribution maps.

The new contribution of this paper is the proposal of monitoring the environmental parameters provided by the heating, ventilation, and air conditioning (HVAC) in a large area of a building by using an autonomous assistant mobile robot [14] equipped with embedded sensors. The environmental information will be used to detect unusual measurements or discrepancies in a conventional indoor working area. The ambient intelligence application will use the information provided by the assistant mobile robot to monitor the environment but using single high performance embedded sensors instead of a distributed network of multiple sensors. In this direction, the assistant mobile robot has the ability to automatically explore and map a wide indoor area and simultaneously gather data of environmental variables such as temperature, air displacement and gas concentration. The data collected with the assistant mobile robot is then used to define an ambient intelligent application that is also able to detect unusual measurements or discrepancies in order to develop further corrective actions.

## 2. Materials and Methods

### 2.1. Assistant Mobile Robot

**Figure 1.**
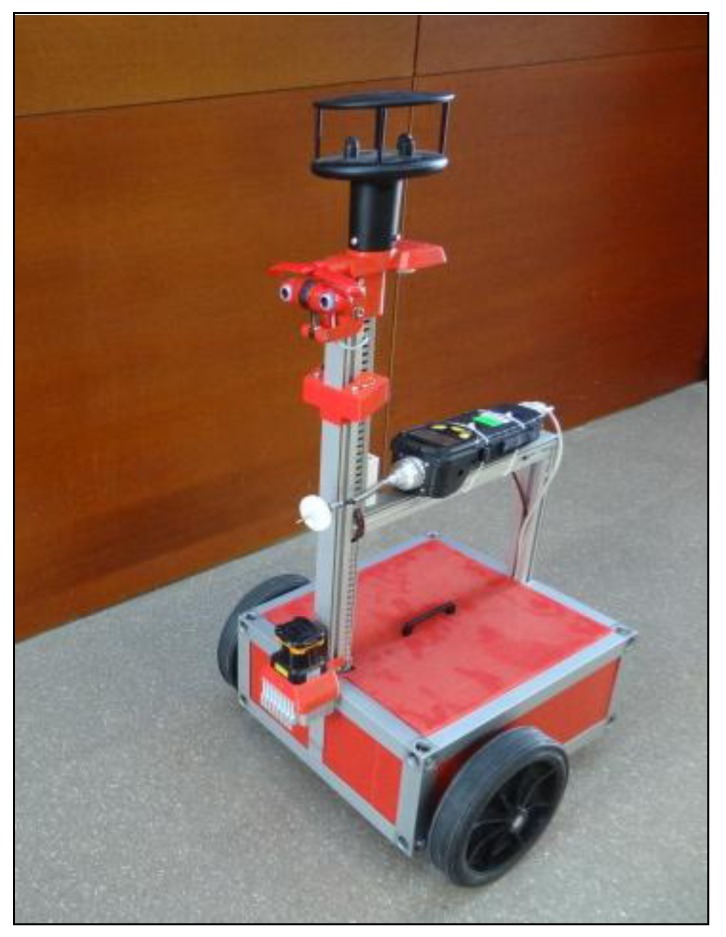
Assistant mobile robot.

Figure 1 shows the assistant mobile robot used in this paper as a mobile measurement agent. The mobile robot agent is implemented in a 1.66 Ghz Intel Core2 duo processor with 1 GB of memory. The mobile robot uses two DC motors (12 V, gear 65:1, model P205 from Micro Motors S.R.L., Verderio Inferiore, Italy) for differential driving; a UTM-30LX USB laser range finder sensor (Hokuyo Automatic CO., LTD, Toyonaka, Japan) with a range of 30 m, 1,080 scan points in an angular range of 270°, and an approximate sampling rate of 4 Hz; a SHT21 I2C temperature sensor (Sensirion, Staefa, Switzerland) with a resolution of 0.01 °C, a sensing accuracy of 0.3% and a sensitivity of 1%; a Windsonic RS232 anemometer by Gill Instruments Inc. (Lymington, UK) that measures the wind velocity in a range up to 60 m/s and the wind direction in degrees; a ppbRAE 3000 RS232 photoionization detector (PID) by RAE Systems (San Jose, CA, USA) configured as an e-nose for gas detection; and a color 3D USB Webcam (2 × 640 × 480 pixels, Minoru, Salford, UK) for additional landmark detection and remote visual control.

### 2.2. Automatic Exploration Procedure

The automatic exploration procedure implemented in this paper requires the utilization of a Simultaneous Location and Mapping (SLAM) methodology on the assistant mobile robot. The implemented SLAM methodology is based on an image template matching method [15] which previously converts the current polar scanning information in one binary image that is compared with the binary image as a representation of the current map of the explored area. This process returns the absolute position of the mobile robot in the map as well as its angular orientation and then the map is updated with the information of the current scanning. The application of this template matching method is limited to a small feasible area of the map and within the range covered by the LIDAR in order to speed up the location procedure.

In this paper, the path planning algorithm implemented in the mobile robot constantly updates the planned mobile robot trajectory in order to explore an indoor area by following the right (or left) wall of the available space while adapting the exploration velocity to the environment. This exploration procedure continues until the assistant mobile robot returns to the starting point or arrives close to a predefined target location. The development of this path planning algorithm combined with the SLAM procedure enables the exploration of all accessible and connected areas in an indoor facility.

### 2.3. Exploration Scenario

**Figure 2.**
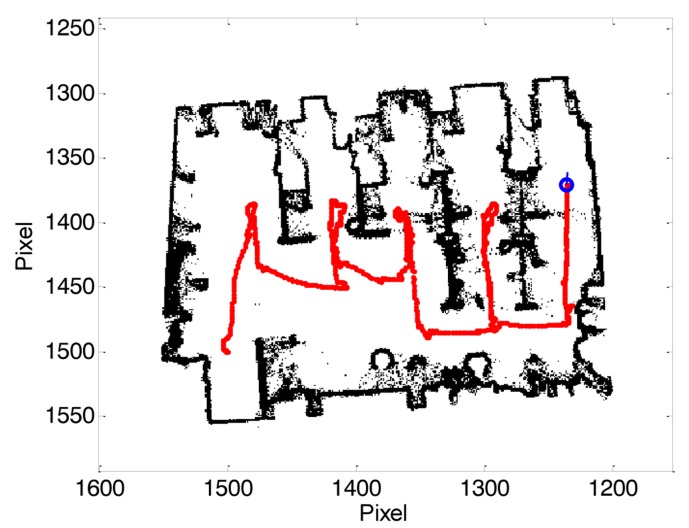
Binary map example obtained in one laboratory, exploration path (red line); robot current position in blue.

The exploration scenario tested in this paper is the second floor of the Polytechnic School of the University of Lleida. This area contains a large central corridor (42 m large and 2.5 m wide) with four laboratories (72 m^2^) in one side and 18 conventional small offices (9 m^2^) in the other side of the corridor. Both, the offices and the laboratories usually have the doors closed when not in use and opened when in use. At this moment, the mobile robot is not able to open a door so the exploration will be limited to the offices and laboratories with the door already open. Therefore, the exploration scenario may change between consecutive explorations. As an example, Figure 2 shows the raw binary map obtained with the SLAM procedure when exploring one of the available laboratories (one pixel of the image is equivalent to 30 mm). In this case, the exploration has started close to the reception desk and ended (blue circle) when the path planning algorithm has detected that all the surface of the laboratory was explored. The noisy points appearing in the raw binary map (Figure 2) can be reduced by performing additional morphological filtering but, in the practice, these noisy pixels have no effect in the SLAM procedure so the computational cost of applying a morphological filtering step after map update can be avoided.

## 3. Ambient Intelligence Application

In this paper, the main goal of the assistant mobile robot is the definition of an ambient intelligence application in the second floor of a research facility that is structured in individual offices and research laboratories. The use of an assistant mobile robot with embedded sensors is designed to obtain similar ambient information than using a distributed network of multiple fixed sensors. In this paper, the mobile robot includes embedded sensors in order to measure temperature, wind speed, and gas concentration. The measurement of temperature and wind speed allows the analysis of the comfort of the different rooms while the measurement of the gas concentration allows the verification of the workplace safety by monitor the concentration of the toxic and non-toxic gases produced and used in the laboratories such as acetone, R-22 (chlorodifluoromethane), and argon-based shielding gasses.

**Figure 3.**
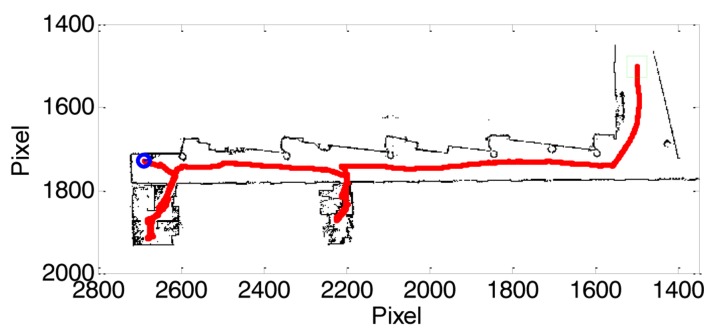
Final map of the facility: starting point (green square), robot path (red line) and ending point (blue circle).

Figure 3 shows an example of the map obtained with the ambient intelligence application and the locations of the sequence of ambientar measurements performed. In this case, the mobile robot started the exploration at the entry hall of the floor (green square in Figure 3) and was configured to explore the complete floor by performing a trajectory close to its left-wall and in front of the offices of the floor. In this exploration example, the assistant mobile robot follows the corridor and enters in two offices which had the door open. The first office was in use and with the HVAC in operation whereas the second office was not in use and the HVAC was switched off. At this moment the assistant mobile robot is not able to enter in a room with a closed door but the complete development of ambient intelligence applications must facilitate this exploration either by using remote operated electronic door locks or adapting the mobile robot to manipulate door handles.

The displacement of the mobile robot inside the small offices requires the realization of small displacements and turns so its average exploring velocity is much slower than when following the open corridor. In this example case, the assistant mobile robot is configured to detect the end of the corridor and stop or pause the current exploration. In the final ambient intelligent application this exploration will continue in order to completely explore both sides of the corridor and return to the starting point.

**Figure 4.**
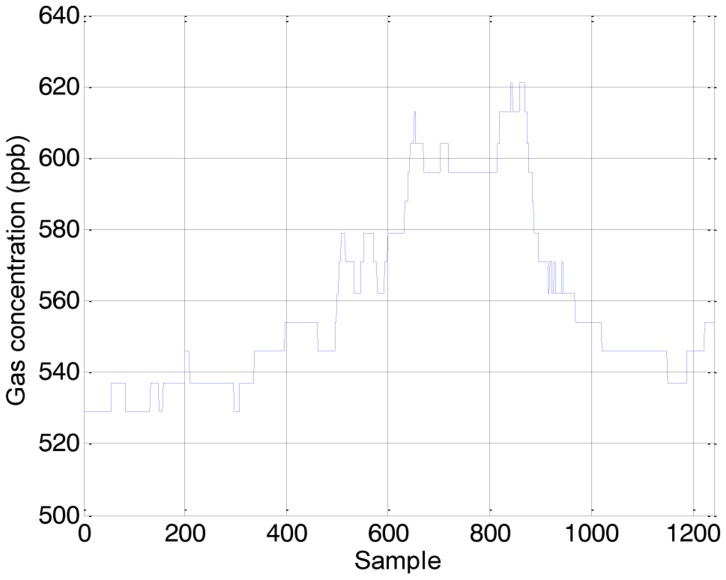
Raw gas concentration samples obtained during the exploration.

**Figure 5.**
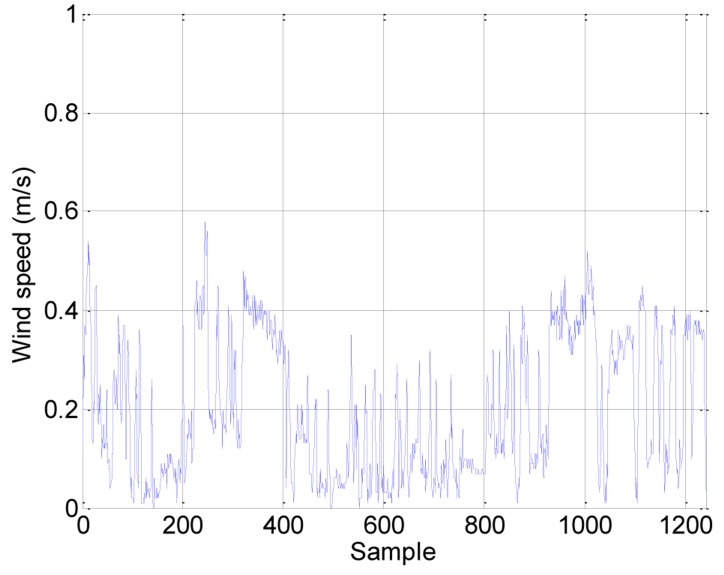
Raw wind speed samples obtained during the exploration.

**Figure 6.**
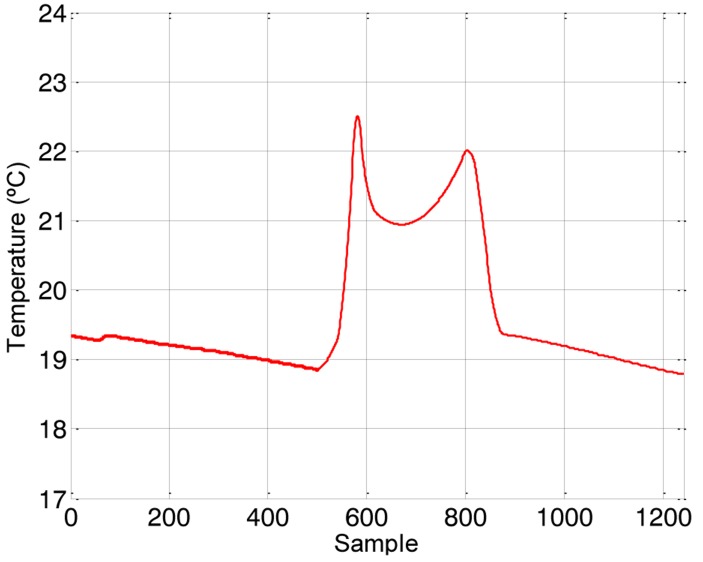
Raw temperature samples obtained during the exploration.

Figures 4, 5 and 6 show the raw data obtained by the sensors embedded in the assistant mobile robot. All the sensors are accessed with the same sampling time. Figure 4 shows the gas concentration measured with the PID sensor configured to detect acetone. The concentrations measured are always below the defined critical security thresholds but the concentration measured has unexpectedly increased inside the first office available and explored. After analyzing this profile, the cause can be the air recirculation system of the building that connects in some way the laboratories and the offices of the same plant. This unexpected connection between the offices and the laboratories can justify the definition of an ambient intelligence application with gas detection capabilities. Figure 5 shows the wind speed measured with the anemometer. The values measured show a constant profile during the complete exploration with peak values lower than 0.6 m/s which is considered as normal.

Finally, Figure 6 shows the temperature profile measured with the assistant mobile robot during the exploration. This temperature profile showed two peaks just when entering and leaving the first explored office (with the HVAC in operation) and an unexpected valley with a reduction of 1.5 °C just in the center of the office which is the occupancy area. This temperature profile has not appeared previously in other explorations that have covered other offices and should originate a warning in the ambient assisted application because a temperature gradients of 1.5 °C in a very small area does not guarantee the occupancy comfort and because most of the energy spent in the HVAC is wasted in non-occupied areas of the office.

## 4. Ambient Actuation

**Figure 7.**
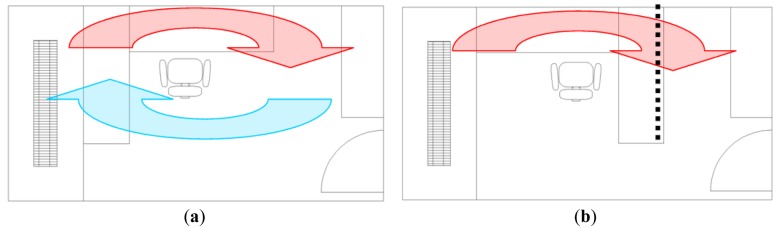
Planned office furniture distribution (**a**); and real furniture distribution of the analyzed office (**b**). The dotted line represents the possible air-blocking effect originated by the table.

The temperature profile obtained during the exploration of the facility has originated an ambient actuation in a specific office of the building. First of all, the efforts have been focused on detecting the cause of the unexpected temperature profile measured. The physical differences with other offices have been carefully analyzed in order to establish some plausible cause hypothesis. Figure 7a shows the furniture distribution planned for the offices; this distribution contributes to the proper circulation of the hot air in the room that is propelled to the ceiling by an axial located below the window as a part of the HVAC. Figure 7b shows a schema of the real furniture distribution in the analyzed office; in this distribution, the table is an obstacle that may block the circulation of the hot air in the room.

**Figure 8.**
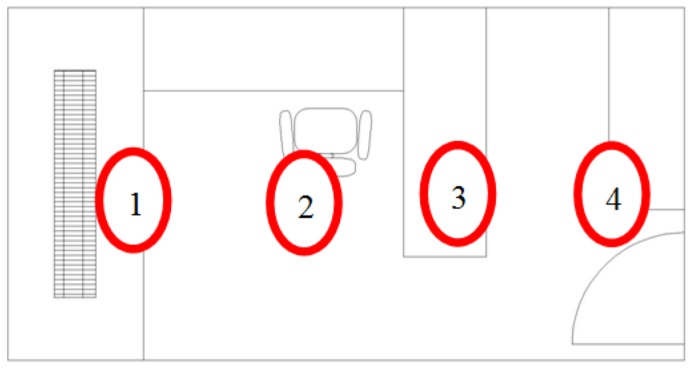
Placement of the sensors for temperature measurement (red marks).

A specific measurement experiment was planned in order to verify the hypothesis that the table is causing an unwanted temperature gradient by blocking the circulation of the hot air in the room. In this experiment four temperature sensors have been placed in four strategic locations in the office (see Figure 8). The measurement point 1 was in the area of the thermostat of the office and also close to the axial fan of the HVAC system. Point 2 was in the occupancy area. The measurement point 3 was over the table of the office. Finally, the measurement point 4 was in the entrance area of the office. The sensors were used to measure the evolution of the temperature approximately during 20 min after switching on the HVAC for the first time at 9 o'clock in the morning. This measurement was repeated several times in order to verify the repetitiveness of the temperature profile obtained.

**Figure 9.**
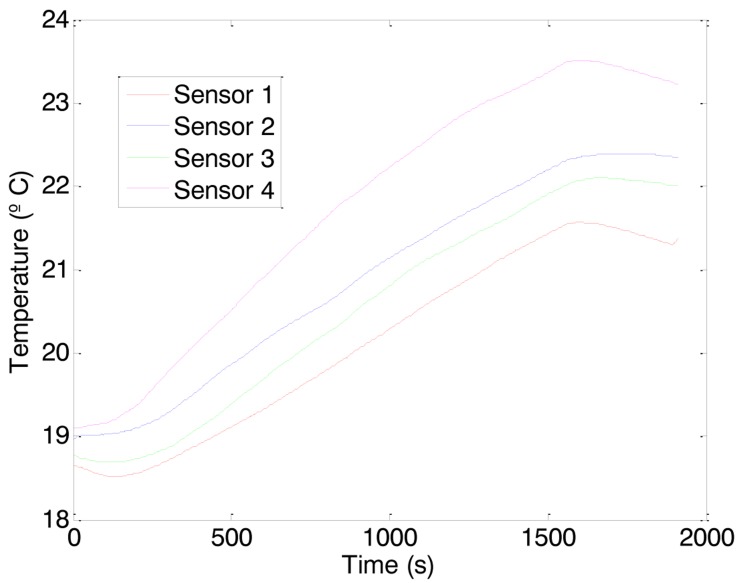
Temperature profile distribution in the office.

Figure 9 shows a representative dynamic temperature profile obtained with the four sensors used. This measurement confirms that the heating effect of the HVAC was focused just in the entrance of the office (Figure 9—Sensor 4). Additionally, the temperature over the table (Figure 9—Sensor 3) was slightly lower than in the occupancy area (Figure 9—Sensor 2) meaning that the propelled hot air arrives to the main occupancy area. This measurement results also reveals that the temperature profile measured in the location of the thermostat (Figure 9—Sensor 1) does not represent the temperature of the occupancy area although it was proportional to the other different temperature profiles obtained in the office.

**Figure 10.**
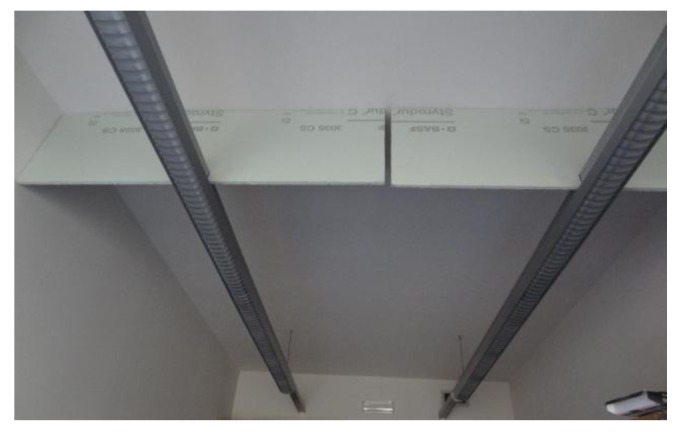
Image of the corrective ambient actuation.

**Figure 11.**
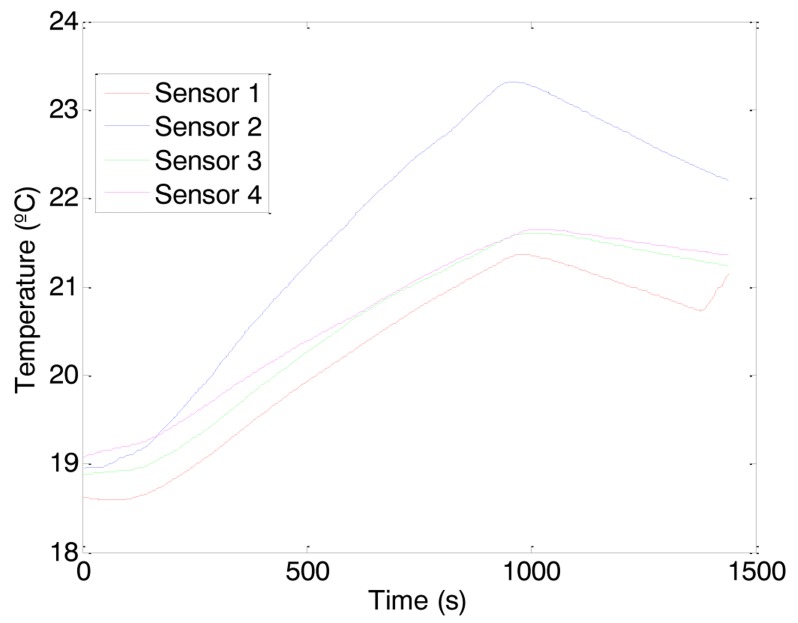
Temperature profile distribution in the office after the ambient actuation.

The temperature profile shown in Figure 9 has been analyzed in order to propose some specific ambient actuations such as: modifying furniture distribution or modifying the path followed by the propelled air. Unfortunately, none of the alternative actuations considered can be implemented or tested by means of electronic actions. Finally, the modification of the furniture distribution was discarded and the manual actuation performed was the placement of a fixed styrofoam panel in the ceiling of the office (Figure 10) in order to block the circulation of the hot air over the occupancy area. Figure 11 shows the new temperature profile obtained in the office after this actuation. In this case, the ceiling trajectory of the hot air was blocked over the occupancy area and this area reaches the highest temperature in the room (Figure 11—Sensor 2). Additionally, the temperature profile over the table (Figure 11—Sensor 3) and next to the entrance door (Figure 11—Sensor 4) was very similar and the temperature profile measured next to the thermostat (Figure 11—Sensor 1) was again proportional to the other different temperature profiles existing in the office. This means that the temperature displayed in the digital thermostat does not represents the real temperature of the occupancy area although there is a direct proportion between them.

## 5. Conclusions

This paper proposes the use of an autonomous assistant mobile robot in order to monitor the environmental conditions of an indoor area as an alternative to the application of a network of fixed environmental sensors. The assistant mobile robot uses onboard sensors to obtain the ambient temperature, the a ir velocity and orientation, and the gas concentration in order to analyze ambient conditions, detect abnormal situations, and propose the development of further ambient actions.

The experimental indoor development of this proposal in one floor of a research facility equipped with offices and laboratories has enabled the detection of an abnormal temperature gradient in one of the offices and the development of a corrective ambient intelligence action in order to improve the occupancy comfort. In this case, the corrective action was the application of a styrofoam panel in the ceiling of the office in order to block the circulation of the air from the HVAC over the occupancy area. The effectiveness of the proposed corrective action was confirmed by means of experimental measurements.

This paper confirms the suitability of an assistant mobile robot that incorporates environmental sensors as a monitoring tool for ambient intelligence applications. The assistant mobile robot is able to measure distributed environmental parameters, detect abnormal ambient situations and improve occupancy comfort and safety. Future works will be focused on estimating energy savings and evaluating occupancy comfort improvement after the application of ambient actions and in the development of accurate predictive temperature models that can be incorporated in the design of improved thermostats in order to improve the human thermal comfort.
